# Oral epithelial dysplasia and aphthous ulceration in a patient with ulcerative colitis: a case report

**DOI:** 10.1186/s12903-023-02851-0

**Published:** 2023-03-11

**Authors:** Kai Sun, Rong-Hui Xia

**Affiliations:** 1 Department of Oral Medicine, Shanghai Ninth People’s Hospital, Shanghai Jiao Tong University School of Medicine, Shanghai, China; 2 College of Stomatology, Shanghai Jiao Tong University, Shanghai, China; 3 National Center for Stomatology, Shanghai, China; 4 National Clinical Research Center for Oral Diseases, Shanghai, China; 5 Shanghai Key Laboratory of Stomatology, Shanghai, China; 6 Department of Oral Pathology, Shanghai Ninth People’s Hospital, Shanghai Jiao Tong University School of Medicine, Shanghai, China

**Keywords:** Aphthous ulceration, Case report, Oral epithelial dysplasia, Ulcerative colitis

## Abstract

**Background:**

Ulcerative colitis is a chronic inflammatory disease with apparent extraintestinal manifestations, including in the oral cavity. Oral epithelial dysplasia, an exclusive histopathological diagnosis that is used to predict malignant transformation, has never been reported with ulcerative colitis. Herein, we report a case with ulcerative colitis that was diagnosed via extraintestinal manifestations of oral epithelial dysplasia and aphthous ulceration.

**Case presentation:**

A 52-year-old male suffering from ulcerative colitis came to our hospital complaining of pain on his tongue with a history of 1 week. Clinical examination revealed multiple painful oval ulcers on the ventral surfaces of the tongue. Histopathological examination indicated ulcerative lesion and mild dysplasia in the adjacent epithelium. Direct immunofluorescence demonstrated negative staining along the junction of the epithelium and lamina propria. Immunohistochemical staining with Ki-67, p16, p53 and podoplanin was used to rule out the reactive cellular atypia to inflammation and ulceration of the mucosa. A diagnosis of aphthous ulceration and oral epithelial dysplasia was made. The patient was treated with mouthwash (composed of lidocaine, gentamicin and dexamethasone) and triamcinolone acetonide oral ointment. Oral ulceration healed after one week of treatment. At the 12-month follow-up, minor scarring was observed on the right ventral surface of the tongue, and the patient felt no discomfort in the oral mucosa.

**Conclusion:**

Oral epithelial dysplasia might also occur in patients with ulcerative colitis despite the low incidence, which should broaden the understanding of oral manifestations of ulcerative colitis.

## Background

Ulcerative colitis (UC) is a chronic inflammatory disease with obscure etiology [1]. The hallmark symptom of the disease is bloody diarrhea [1]. Although UC primarily involves the bowel, extraintestinal manifestations of the disease are often apparent. Oral signs of UC include specific pyostomatitis vegetans and non-specific aphthous ulceration, angular cheilitis, gingivitis and periodontitis [2, 3]. The most common oral manifestation is aphthous ulceration, which is benign and occurs in 20% of patients with UC [4].

Oral epithelial dysplasia (OED) is an exclusive histopathological diagnosis that involves a group of oral potentially malignant disorders, including oral leukoplakia, oral lichenoid lesions and oral submucous fibrosis, and is used to predict the malignant transformation of these disorders [5]. In patients with UC, epithelial dysplasia is reported to be limited to the colon and rectum [6], while epithelial dysplasia in the oral cavity has never been reported [3].

We report a case of UC accompanied by aphthous ulceration and OED, which should broaden the understanding of oral manifestations of UC. This case report was approved by the Ethics Committee of Shanghai Ninth People’s Hospital, Shanghai Jiao Tong University School of Medicine (SH9H-2022-T132-2). Written informed consent was obtained from the patient according to the Declaration of Helsinki.

## Case presentation

A 52-year-old male presented to the Department of Oral Medicine, Shanghai Ninth People’s Hospital for pain in his tongue with a history of one week. The patient had been experiencing recurrent oral pain for nearly two years and reported that he had not used any medication for his oral pain. The pain would disappear spontaneously after two weeks and then recur within ten days, which affected the patient’s daily life. Dermatological or genital discomfort or lesions were negative. The patient was diagnosed with UC 5 years ago and mesalazine had been prescribed. Other systemic diseases were negative except for lacunar infarction of the brainstem, which was diagnosed six months ago. The patient had a family history of recurrent aphthous ulceration. Smoking and alcohol consumption were negative.

**Fig. 1:**
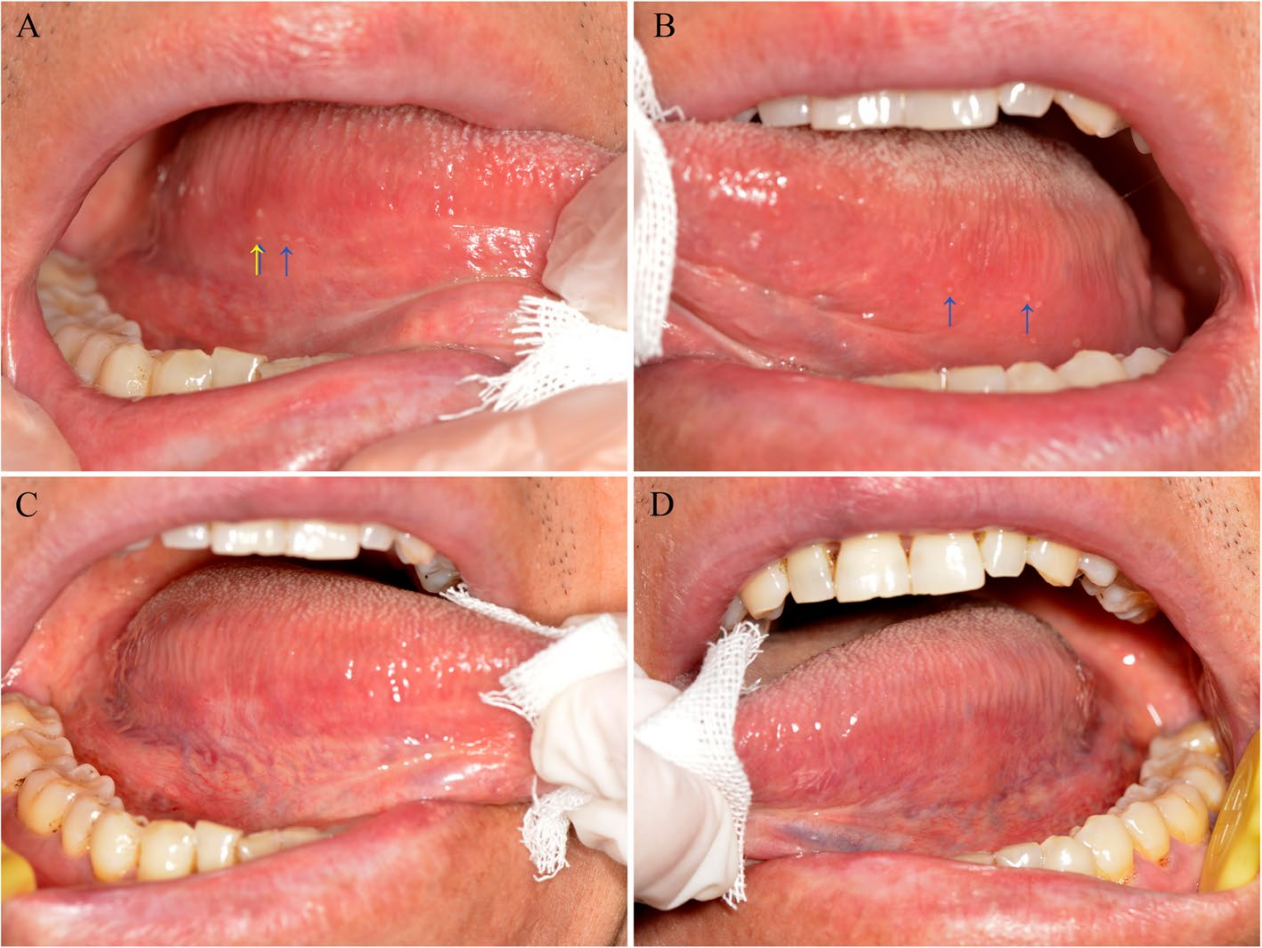
Clinical manifestations of oral mucosa before and after treatment. (**A**) – (**B**) Aphthous ulceration before biopsy and treatment; **A** right ventral surface of the tongue, blue arrows indicate the oral ulcers and yellow arrow indicates the region of biopsy; **B** left ventral surface of the tongue. (**C**) – (**D**) Oral manifestations at one-year follow-up; **C** right ventral surface of the tongue and **D** left ventral surface of the tongue

**Fig. 2:**
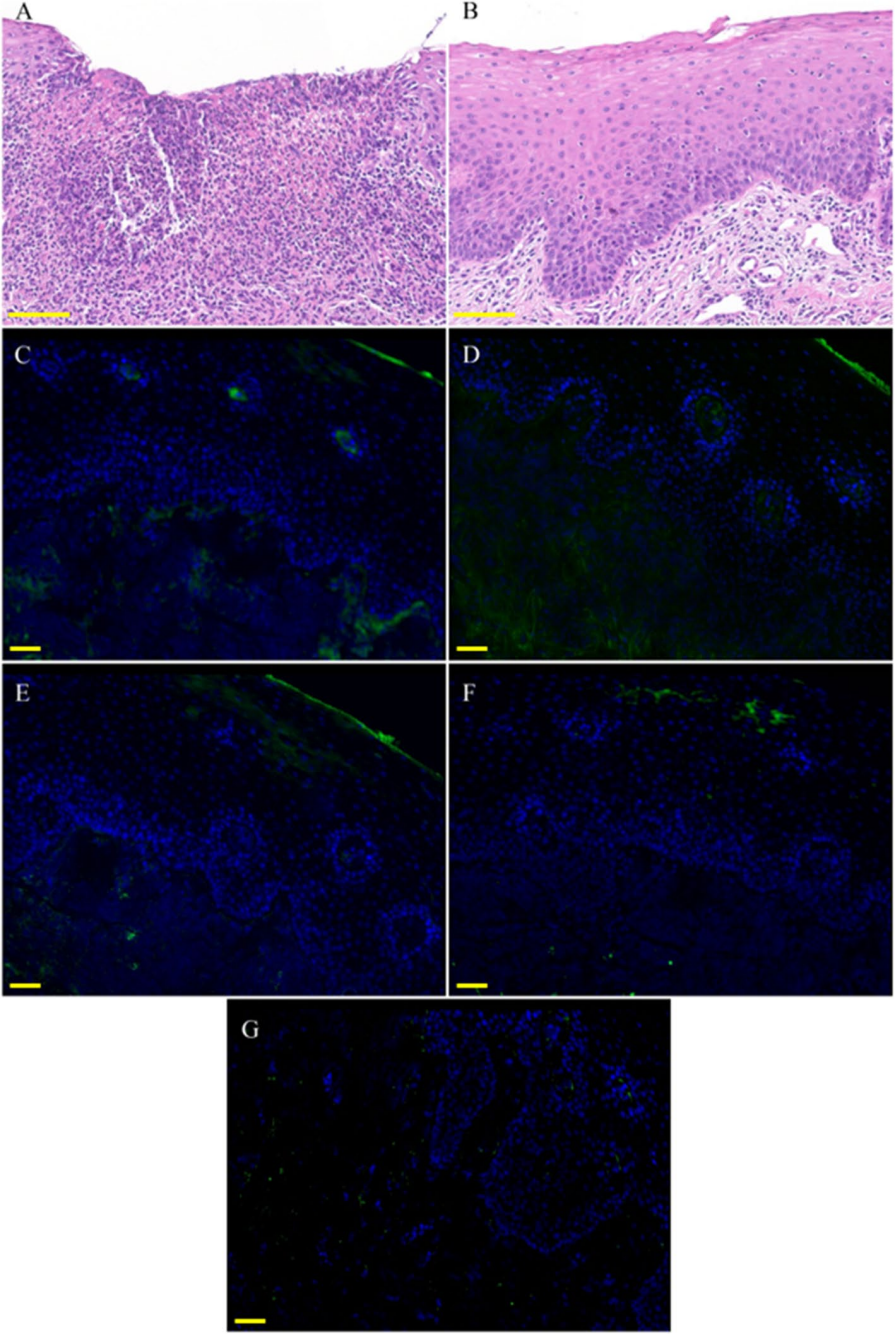
Histopathological examination of the oral lesion. (**A**) – (**B**) Hematoxylin and eosin, 200 × ; **A** an ulcerative lesion with chronic inflammation. **B** the epithelium displayed mild dysplasia. (**C**)—(**G**) Direct immunofluorescence; **C** C3; **D** fibrinogen; **E** IgA; **F** IgM; and **G** IgG; Ig, immunoglobulin. Scale bar: 100 μm

**Fig. 3:**
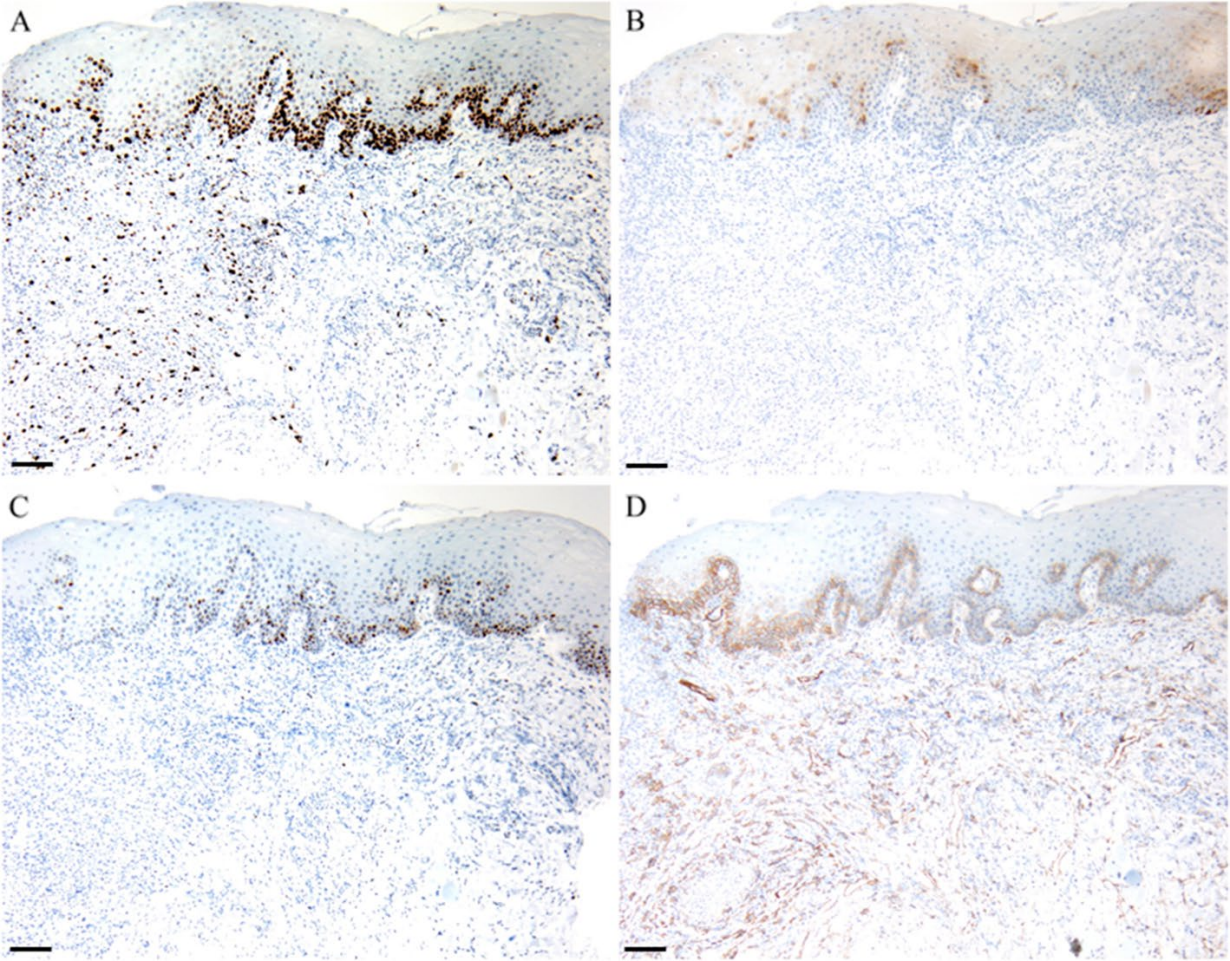
Immunohistochemical staining of the oral lesion. **A** Ki-67. **B** P16. **C** P53. **D** Podoplanin. Scale bar: 100 μm

Clinical examination demonstrated multiple painful oval ulcers with a yellow pseudomembranous base and erythematous borders on the bilateral ventral surfaces of the tongue (Fig. 1). The ulcers were 1 – 2 mm in diameter, and the texture was homogeneously soft. No other oral or dermatological lesions were observed. Laboratory tests revealed decreased levels of red blood cells, hemoglobin, mean corpuscular hemoglobin, and mean corpuscular hemoglobin concentration and increased levels of fibrinogen and C-reactive protein. Autoimmune bullous disease antibodies and routine clinical biochemistry indexes were within the normal range. Fecal occult blood testing and fecal transferrin were positive (Table 1). An electronic colonoscopy biopsy depicted chronic and active rectal inflammation with erosion. To confirm the diagnosis of the oral lesions, a biopsy was performed on the right ventral surface of the tongue. Histopathological examination by two independent oral pathologists indicated chronic mucosa inflammation, with inflammatory cell infiltration, vascular proliferation, and focal ulcer formation in the subepithelial region. Mild dysplasia was diagnosed based on architectural and cytological features of the 2017 World Health Organization classification, which included irregular epithelial stratification, loss of polarity of basal cells, drop-shaped rete ridges, increased number of mitotic figures, abnormally superficial mitoses, premature keratinization in single cells (dyskeratosis), keratin pearls within rete ridges, loss of epithelial cell cohesion, abnormal variation in nuclear size, abnormal variation in nuclear shape, abnormal variation in cell size, abnormal variation in cell shape, increased nuclear-cytoplasmic ratio, atypical mitotic figures, increased number and size of nucleoli, and hyperchromasia (Fig. 2A-B). Direct immunofluorescence demonstrated negative staining for fibrinogen, immunoglobulin G (IgG), IgA, IgM, and C3 along the junction of the epithelium and lamina propria (Fig. 2C-G). Furtherly, immunohistochemical staining with Ki-67, p16, p53 and podoplanin was used to rule out the reactive cellular atypia to inflammation and ulceration of the mucosa. Ki-67 expression in the suprabasal layer was detected; p53 expression was detected in the basal layer; p16 expression was detected in few basal cells; podoplanin was positively expressed in basal and suprabasal layer of the epithelium (Fig. 3).

**Table 1:** Results of laboratory tests of the patient

Indexes	Results	Normal range
Red blood cell	4.04 × 10^12^/L	4.3–5.8 × 10^12^/L
Hemoglobin	102 g/L	130–175 g/L
Mean corpuscular hemoglobin	25.2 pg	27–34 pg
Mean corpuscular hemoglobin concentration	304 g/L	316–354 g/L
Fibrinogen	5.4 g/L	1.8–3.5 g/L
C-reactive protein	23.36 mg/L	0–8 mg/L
Autoimmune bullous dermatosis antibody	negative	negative
Total bilirubin	5.3 µmol/L	0–23 µmol/L
Alkaline phosphatase	64 U/L	45–125 U.L
γ-glutamyl transferase	12 U/L	10–60 U.L
Alanine aminotransferase	9.5 U/L	9–60 U.L
Aspartate aminotransferase	16.9 U/L	15–45 U/L
Creatinine	68.0 µmol/L	57–97 µmol/L
Fecal occult blood testing	positive	negative
Fecal transferrin	positive	negative

The diagnosis of aphthous ulceration and OED was made based on clinical and histopathological examinations. The patient was treated with mouthwash (composed of 100 mg lidocaine, 160 mg gentamicin, and 5 mg dexamethasone in 200 mL of normal saline) and 0.1% (w/w) triamcinolone acetonide oral ointment three times a day. The oral ulceration healed without adverse or unanticipated events after one week of treatment. The patient was suggested to treat anemia. Close surveillance of systemic and oral diseases and a follow-up interval of six months were also recommended to the patient. At the 6-month follow-up by telephone, the patient reported that he had not suffered any pain in his oral mucosa, and no abnormality was noted on the biopsy area. At the 12-month follow-up, minor scarring was observed on the right ventral surface of the tongue (Fig. 1C-D), and the patient felt no discomfort in the oral mucosa. The patient was satisfied with the effect of the treatment.

## Discussion and conclusions

This report presents a case of UC that was diagnosed extraintestinally as aphthous ulceration and OED. To our knowledge, this is the first time that OED has been reported in patients with UC. Our case could provide some clues for managing oral lesions in UC patients.

Oral epithelial dysplasia is an exclusively histopathological diagnosis that is rendered for a spectrum of diseases [7], with an overall prevalence of 5.71% [8]. In a recent study, 552 cases (57.9%) with oral leukoplakia, leukoerythroplakia, erythroplakia, or actinic cheilitis exhibited OED [9], which indicates that OED may be a non-specific extraintestinal manifestations of UC. The molecular mechanism of OED remains poorly understood; however, the presence of OED usually indicates a risk of malignant transformation [7]. The factors of severe dysplasia, large size, nonhomogeneous texture and red color are strongly associated with malignant transformation [10]. In this case, the patient was classified as low-risk potential based on the clinical and histopathological examination, and no further treatment was implemented. Since patients with UC are at higher risk of developing colorectal cancer, cholangiocarcinoma, liver-biliary tract cancer and leukemia than the general population [11], close surveillance of systemic and oral diseases and regular follow-ups were recommended.

In our case, a panel of biomarkers, including Ki-67, p16, p53 and podoplanin, was detected to differentiate genuine epithelial dysplasia from reactive cellular atypia. Ki-67 acts as a more sensitive and specific marker than histopathological analysis of mitoses, nuclear pleomorphism, and increased nuclear cytoplasmic ratio [7, 12]. The Ki-67 expression was detected in the basal and parabasal layers of normal oral mucosa, and increased according to the grade of dysplasia in the suprabasal layer [13]. Immunohistochemistry for p16 with a threshold of 70% positive cell staining correlates well with the diagnosis of human papillomavirus-associated dysplasia [7]. The function of p53 in the prediction of malignant transformation has been focused as p53 is an early alteration in oral dysplastic lesions [14]. Podoplanin is associated with tumor development in an oral dysplasia‐carcinoma sequence through clonal expansion of stem cells [15]. The podoplanin immunohistochemical staining was absent in the normal oral mucosa [16], and its expression pattern correlates with degree of dysplasia in a grade‐dependent manner [15, 17]. Taken together, the expression pattern of these biomarkers in this case indicated that reactive atypia could be ruled out.

In addition to OED, aphthous ulceration was observed in this case, which has previously always been non-specific in UC patients. Epidemiologically, there is no difference in the prevalence of aphthous ulceration in UC patients and the general population [18]. The relationship between aphthous ulceration and the activity phase of UC is still unclear [3]. It is reported that aphthous ulceration in UC patients may be caused by malnutrition owing to intestinal malabsorption and rectal bleeding and by drugs used for UC treatment due to their direct toxic or indirect immunosuppressive effects. In this case, anemia and mesalazine might have resulted in aphthous ulceration. Management of anemia was suggested to the patient. In addition, we prescribed corticosteroids, topical anesthetics, and antiseptic mouthwash to the patient, and excellent clinical results were achieved.

There are some limitations associated with this case. First, the follow-up period for this case was relatively short, which made it difficult to evaluate the long-term prognosis of OED. Second, unlike pyostomatitis vegetans, OED might be non-specific to UC. The prevalence of OED in UC patients needs to be studied in the future.

In conclusion, we reported a case of UC with aphthous ulceration and OED, indicating that OED might also occur in patients with UC despite the low incidence. Close surveillance of systemic and oral diseases and regular follow-ups are recommended for patients with oral manifestations of UC.

## Data Availability

The datasets used and/or analyzed during the current study are available from the corresponding author upon reasonable request.

## Abbreviations

UC: Ulcerative Colitis
OED: Oral Epithelial Dysplasia
Ig: Immunoglobulin

## References

[1] Gajendran M, Loganathan P, Jimenez G, Catinella AP, Ng N, Umapathy C, Ziade N, Hashash JG (2019). A comprehensive review and update on ulcerative colitis. Dis Mon.

[2] Tan CX, Brand HS, de Boer NK, Forouzanfar T (2017). Gastrointestinal diseases and their oro-dental manifestations: Part 2: Ulcerative colitis. Br Dent J.

[3] Lauritano D, Boccalari E, Di Stasio D, Della Vella F, Carinci F, Lucchese A, Petruzzi M (2019). Prevalence of oral lesions and correlation with intestinal symptoms of inflammatory bowel disease: a systematic review. Diagnostics (Basel).

[4] Elahi M, Telkabadi M, Samadi V, Vakili H (2012). Association of oral manifestations with ulcerative colitis. Gastroenterol Hepatol Bed Bench.

[5] Tilakaratne  WM, Jayasooriya  PR , Jayasuriya  NS, De Silva  RK (2019). Oral epithelial dysplasia: causes, quantification, prognosis, and management challenges. Periodontol 2000.

[6] von Herbay A, Herfarth C, Otto HF (1994). Cancer and dysplasia in ulcerative colitis: a histologic study of 301 surgical specimen. Z Gastroenterol.

[7] Odell E, Kujan O, Warnakulasuriya S, Sloan P (2021). Oral epithelial dysplasia: recognition, grading and clinical significance. Oral Dis.

[8] Singh S, Singh J, Chandra S, Samadi FM (2020). Prevalence of oral cancer and oral epithelial dysplasia among North Indian population: a retrospective institutional study. J Oral Maxillofac Pathol.

[9] de Azevedo AB, Dos Santos T, Lopes MA, Pires FR (2021). Oral leukoplakia, leukoerythroplakia, erythroplakia and actinic cheilitis: analysis of 953 patients focusing on oral epithelial dysplasia. J Oral Pathol Med.

[10] Speight PM, Khurram SA, Kujan O (2018). Oral potentially malignant disorders: risk of progression to malignancy. Oral Surg Oral Med Oral Pathol Oral Radiol.

[11] Annese V, Beaugerie L, Egan L, Biancone L, Bolling C, Brandts C, Dierickx D, Dummer R, Fiorino G, Gornet JM (2015). European evidence-based consensus: inflammatory bowel disease and malignancies. J Crohns Colitis.

[12] Tabor M, Braakhuis B, van der Wal J, van Diest P, Leemans C, Brakenhoff R, Kummer J (2003). Comparative molecular and histological grading of epithelial dysplasia of the oral cavity and the oropharynx. J Pathol.

[13] Takeda T, Sugihara K, Hirayama Y, Hirano M, Tanuma J, Semba I (2006). Immunohistological evaluation of Ki-67, p63, CK19 and p53 expression in oral epithelial dysplasias. J Oral Pathol Med.

[14] Braakhuis BJ, Tabor MP, Kummer JA, Leemans CR, Brakenhoff RH (2003). A genetic explanation of Slaughter's concept of field cancerization: evidence and clinical implications. Cancer Res.

[15] Inoue H, Miyazaki Y, Kikuchi K, Yoshida N, Ide F, Ohmori Y, Tomomura A, Sakashita H, Kusama K (2012). Podoplanin expression during dysplasia-carcinoma sequence in the oral cavity. Tumour Biol.

[16] Lunawat S, Prakash N, Pradeep G, Chaware S, Chaudhari N, Salunkhe V (2021). Assessment of podoplanin lymphatic vessel density in oral epithelial dysplasia. J Oral Maxillofac Pathol.

[17] Verma V, Chandrashekar C (2019). Evaluation of SOX2 and podoplanin expression in oral epithelial dysplasia and its correlation with malignant transformation. J Investig Clin Dent.

[18] Thrash B, Patel M, Shah KR, Boland CR, Menter A. Cutaneous manifestations of gastrointestinal disease: part II. J Am Acad Dermatol. 2013; 68(2): 211 e1–33; quiz 44–6. 10.1016/j.jaad.2012.10.037 23317980

